# A secure and dependable trust assessment (SDTS) scheme for industrial communication networks

**DOI:** 10.1038/s41598-023-28721-x

**Published:** 2023-02-02

**Authors:** Tayyab Khan, Karan Singh, Khaleel Ahmad, Khairol Amali Bin Ahmad

**Affiliations:** 1 Jawaharlal Nehru University, New Delhi, India; 2 Department of Computer Science and Information Technology, Maulana Azad National Urdu University, Hyderabad, Telangana India; 3 Department of Electrical and Electronic Engineering, Faculty of Engineering, National Defence University of Malaysia, Kuala Lumpur, Malaysia

**Keywords:** Engineering, Mathematics and computing

## Abstract

Due to tamper-resistant sensor nodes and wireless media, Industrial Wireless Sensor Networks (WSNs) are susceptible to various security threats that severely affect industrial/business applications. The survival of sensor networks is highly dependent on the flourishing collaboration of sensor nodes. Trust management schemes seem to be realistic and promising techniques to improve security as well as cooperation (dependability) among sensor nodes by estimating the trust level (score) of individual sensor nodes. This research paper presents a well-organized and motivating secure, dependable trust assessment (SDTS) scheme for industrial WSNs to cope with unexpected behavior such as an on–off attack, bad-mouthing attack, garnished attack, etc., by employing robust trust evaluation components based on success ratio and node misbehaviour. SDTS incorporates an interesting trust evaluation function in which the trust range can be adjusted in accordance with the application requirement. SDTS include direct communication trust, indirect communication trust, data trust, and misbehavior-based trust to defend the multiple internal attacks. SDTS works according to the behavior of nodes, i.e., whether the sensor nodes are interacting frequently or not. Moreover, abnormal attenuation and dynamic slide lengths are incorporated in the proposed model (SDTS) to deal with various natural calamities and internal attacks. SDTS is compared against three recent state-of-the-art methods and found efficient in terms of ease of trust assessment, false-positive rate (2.5%), false-negative rate (2%), attack detection rate (90%), detection accuracy (91%), average energy consumption (0.40 J), and throughput (108 Kbps) under the load of 500 sensor nodes with 50% malicious nodes. Investigational results exhibit the potency of the proposed scheme.

## Introduction

A wireless sensor network (WSN) consists of low-cost, spatially distributed sensor nodes (SNs), forming a low-level self-organized network. SNs avoid complex cabling and provide a flexible solution for various applications such as industrial applications for continuous sensing and remote monitoring, controlling different industrial parameters, monitoring system status, and machine health^1^. It plays a vital role in several other applications such as traffic control, process control, environmental monitoring, smart roads, automation, automotive, homeland security, public security, healthcare applications, workplaces (job, office), and even in aircraft as well as in many critical applications such as defense surveillance applications, target tracking, transportation systems, security, home applications, cyber-physical system (CPS) security in terms of detection, real-time monitoring, enhancing efficiency, reduce safety cost as well as save labor. SNs have limited resources (processor, memory, battery, communication bandwidth) which periodically send the sensed data gathered through wireless links to the base station (BS). Then, the BS processes as well as analyze the critical data and sends it to the end-user for decision-making, including controlling and monitoring. The base station (gateway or sink) is the core node responsible for coordinating the network and centralizing all system signals. It collects the monitored data, processes it, and stores it in the information management system. Moreover, the sink is capable of retransmitting the sensed information to the external world. The survival of WSNs highly depends on the mutual and sincere cooperation of SNs to achieve high network performance such as high throughput and efficiency of routing traffic towards the base station^2^. Nowadays, WSNs are mature enough to be used by industrial applications, business applications, and tracking applications that rely on trustworthy information provided by SNs to control the running process^3^. The growth and reputation of the industry entirely rely on the reliability of data observed by the resource constraints SNs, hence the industrial communication networks (ICNs) must ensure the data availability, confidentiality, and authenticity. ICNs are essentially industrial WSNs that monitor the various industrial activities of industrial plants^4^. An efficient architecture of resource constraints ICN offers a variety of benefits in terms of energy efficiency, scalability, and reliability. ICNs strongly require a trustworthy assessment tool to check and analyze the reliability of SNs data since the monitored or delivered data is used in decision-making^5^ by the industries for their growth as well as survival.

The primary objective of dependability (trustworthiness) evaluation is to assist industrial or business applications for accurate decision-making as well as distinguish reliable and erroneous data from the monitored data^6^. An incorrect decision based on the observed behavior of SNs severely influences the growth and reputation of any industry/business in the real world^7^. Trustworthy assessment models (TAM) in industrial applications provide a lot of significant benefits^8^. ICN nodes themselves lack tamper-resistant hardware and are vulnerable to diverse internal and external security threats (attacks). The SNs can be captured physically by attackers. Due to its deployment nature (which includes free and unprotected communication channels, hostile environment, severe constraints, and topology), cryptographic techniques like authentication, encryption, decryption, SUBBASE, LEAP, SNEP, ECC, etc. impose a high cost, overheads as well as power consumption and hence, failed to detect and mitigate spiteful behavior caused by faulty SNs^9^. These misbehaviors are known as internal attacks and are categorized into multiple categories based on the unexpected behavior of SNs such as on–off, sinkhole, black hole, Wormhole attack, Sybil attack, Spoofing attack, etc. These internal attacks may cause delay, transmission failure, link failure, and massive economic loss in industries. One significant, powerful, cost-efficient, and adaptive security solution (tool) for such problems is the trust assessment model (TAM). TAM provides an alternative, practical, robust, and reliable solution to detect and mitigate such types of internal adversaries and abnormal behaviors caused by internal malicious nodes^10^. TAM estimates the communication and data trust score of SNs by considering the monitored behavior (interactions) and makes a suitable decision in terms of reliability or faulty^11^. Furthermore, TAM updates trust to provide security and enhance dependability. Each SN acts as a router to forward generated traffic toward its neighbors or sink node^12^. Table 1 defines the various terminologies related to trust.

**Table 1:** Various terminologies related to trust.

Various terminology related to trust	
Terminology	Definition
Trust (expectation)^6^	Level of assurance or confidence of an SN x that SN y will perform as expected
Trust value^20,27^	Trust value is a level (quantification or measure) of belief of one entity (SN) towards another entity (SN). It is denoted by numerical value within a specified range
Trust evaluation^8^	Monitor the behavior, estimate the trust value and then quantify it into highly trusted, trusted, distrusted, etc
Trust model^21^	Methods to estimate trust scores and decide the trustworthiness level of SNs are known as trust models
Trust management scheme^5,11^	Schemes to monitor the behavior of SNs, compute their trust, and update it periodically
Direct trust^14,17^	Individual and independent belief of one entity towards another entity in terms of trustworthiness. Not symmetric
Indirect trust (feedback trustor, Recommendation trust or reputation)^18,19,21^	Level of assurance (trustworthiness or belief) on an intermediate node (entity) about another entity
	OR The information (trust score) provided by a node y to node x about node c is called reputation (indirect evidence)
Energy trust^7,16^	Level of assurance about battery power residual to perform tasks in an appropriate way

From the above discussion, it is clear that the survival of industrial WSN (ICN) depends on the accuracy of the sensed data, which is further dependent on the trust model. A robust and flexible TAM must ensure efficient industrial safety quality and competent monitoring operations in different environments^17^. Moreover, TAM must be feasible, and secure, impose minimal overheads as well as validate the functional requirements^20^. To remove the mentioned limitations of ICMs, the proposed Trust assessment model (SDTS) consist of several unique characteristics as listed below:

- No single point of failure because of the hybrid approach. It employs an innovative trust function, which punishes and rewards malicious and genuine nodes, respectively.
- It employs direct communication trust, data trust, indirect trust, and current and past misbehavior, weight, and frequency of misbehavior^13,14^. It is unique in terms of its trust computation process, trust evaluation criteria, elimination of untrustworthy SNs, and assigning appropriate weightage since trust calculation based on success ratio reduces power consumption.
- Effectively defend against severe attacks such as on–off, bad-mouthing, grey hole, garnished, selective forwarding, and whitewashing attacks. It exhibits superior performance in terms of detection, mitigation, computational overhead, and processing overhead.
- Well suited for real-time critical industrial applications. Moreover, it explores trust metrics, unexpected behaviors, information collection and dissemination, the implication of trust models, design strategies, etc.

This paper recommends a secure and dependable trust assessment (SDTS) scheme for ICNs. Firstly, we discuss the architecture of trust assessment based on the characteristics of ICNs. Secondly, we highlight the importance of the optimal number of clusters in IWSNs (ICNs) and find the optimal number of clusters. Thirdly, a trust assessment model is projected to estimate the trust score of SNs by considering the effect of the centralized security manager. Trust evaluation is done for each case (1) when nodes are frequently interacting, and (2) when nodes are not interacting frequently. For the first case, we employ a cooperative interaction-based trust evaluation method that incorporates direct communication trust, data trust, and indirect trust. Moreover, we observe the deviation degree of indirect trust using our previous work^13^ to filter bogus evaluations from malicious nodes. Furthermore, we incorporate a dynamic length sliding window concept^13^ to monitor the good and bad behaviors of SNs to remove the limitation of the fixed-length sliding window. In addition, we suggest a trust-based malicious node detection algorithm to detect various kinds of internal attacks. For the second case, when nodes are not interacting frequently, we incorporate a misbehavior component proposed in our previous work^14^ during trust evaluation. The misbehavior component includes current and past misbehavior, weight, and frequency of misbehavior in the past and present. Then, we provide the absolute trust score based on the misbehavior component. Finally, we compare SDTS with some classic trust assessment schemes in the simulations. The results show that SDTS can improve trust assessment severity, accuracy, and detection rate in harsh industrial environments and make the WSN more secure.

The remaining part of this paper is organized as follows: Section “Introduction” presents a concise overview of trust and related terminologies. Section “Literature review” presents a comprehensive overview of the related works. In this section describes trust management, the optimal number of clusters, and the trust evaluation model in IWSNs. Section “Simulation and result analysis” discusses the simulation results. Lastly, section “Conclusion and future direction” winds up the entire paper and suggests future work.

## Literature review

This section discusses the methodology, advantages, and limitations of the various existing state-of-the-art trust models. It has been observed that most of the TMSs do not focus on the fundamental requirements of industrial WSNs (ICNs). Table 2 summarizes the existing trust models in terms of various parameters and limitations.

**Table 2:** Comparative study of recent trust models.

Trust management Scheme	Observation	Abnormal attenuation factor	Adjustable Trust range	Dynamic slide length	Limitation
LDTS^17^	Only communication trust is incorporated in the trust function	Not considered during trust evaluation	No	No	Incorporation of data trust and energy trust is neglected, Static penalty coefficient so susceptible to various internal attacks
GTMS^18^	Only communication trust is incorporated in the trust function	Not considered during trust evaluation	No	No	Linear and static trust function, not robust to detect internal attacks, high complexity, weak penalty to selfish nodes
LWTM^20^	Only communication trust is incorporated in the trust function	Not considered during trust evaluation	No	No	Suitable for 1-hop clustered WSN, not robust against on–off attack, Sybil attack, less responsive to other attacks
ADCT^21^	Communication and Data trust are included in the trust function	Not considered during trust evaluation	No	No	Non-adaptive trust function, suitable for limited attacks, decision making without considering the dynamic cluster group
ETRES^22^	Only direct and indirect trust based on the exponential distribution	Not considered during trust evaluation	No	No	Exponential trust function, punishment, and reward strategy is not flexible, suitable for only on–off and collusion attack detection
^23^	Multi-trust (communication trust, data trust, and energy trust) scheme provides a secure CH election algorithm and a misbehavior detection approach	Not considered during trust evaluation	No	No	High computational overhead, punishment, and reward is not dynamic with interactions of SNs
^24^	Only behavioral-based trust, direct and indirect trust, and data-based trust is composite during trust evaluation	Not considered during trust evaluation	No	No	Effect of malicious nodes on trust value is ignored, communication overhead and memory overhead is not discussed, not robust against on–off and Sybil attacks
BTEM^25^	Only direct and indirect trust is employed, data trust is ignored, isolate malicious nodes, enhance the false-positive detection rate	Not considered during trust evaluation	No	No	Robust against only Bad-mouth, On–Off, and Denial of Service (DoS) attacks, not incorporate reward and punishment to good and bad nodes, respectively
LTS^29^	Communication and Data trust with flexible punishment coefficient	Not considered during trust evaluation	No	No	Computation of Success ratio does not include beta function, suitable for limited attacks such as a garnished attack, blackhole attack, and Bad mouthing attack
ETS^30^	Only direct communication trust, energy trust	Not considered during trust evaluation	No	No	Punishment and reward is not flexible, Not resilient against on–off attack, does not incorporate indirect trust and data trust, moderate complexity, not able to detect a small percentage of misbehavior due to Sybil attack
BDTM^31^	Only direct and indirect communication trust	Not considered during trust evaluation	No	No	Not robust against Sybil, collusion attack. Not able to detect a small percentage of misbehavior due to On–Off attack, static trust function
BLTM^32^	Communication trust, Data trust, and energy trust	Not considered during trust evaluation	No	No	Linear trust function, punishment, and reward strategy is not flexible, not robust against on–off and badmouthing attacks, not able to detect a small percentage of misbehavior, quadratic computational complexity
TMA^33^	Only communication trust is incorporated in the trust function	Not considered during trust evaluation	No	No	Linear and static trust function, not robust to detect internal attacks such as DoS attack, Sybil attack etc
^39–45^	Data trust is ignored	Not considered during trust evaluation	No	No	Complex trust functions, not robust against internal adversaries, unsatisfactory performance with more malicious nodes, consume more energy
^51^	Entropy-based direct opinion and indirect opinion trust computation	Not considered during trust evaluation	No	No	Suitable for distributed hashing with minimal time delay, not robust to detect internal attacks
^52^	Maintain age and freshness of information, minimize EWSA	Not considered during trust evaluation	No	No	not robust to detect internal attacks, only improve AoI and minimize EWSA
SDTS [proposed method]	Optimal number of clusters, Direct Communication trust, indirect communication trust with deviation degree, Data trust, dynamic slide length of logical time window	Considered during trust evaluation	Yes	Yes	NA

In Gomez et al.^8^, proposed a trustworthiness estimation scheme for business applications and discussed the impact of trust models on industrial applications, their growth, and survival. Moreover, a subjective logic approach is used in the trust assessment of routed and processed sensor data. In Li et al.^17^, suggested that a cluster-based lightweight TMS (LDTS) improves system efficiency and dependability by alleviating the compromised nodes. LDTS utilizes a self-adaptive weighted concept at cluster head for trust score aggregation. However, LDTS does not employ data trust and energy trust and is not robust against an on–off attack. Moreover, it is not scalable and uses a static punishment coefficient that makes it unrealistic for industrial/business applications. In Shaikh et al.^18^, designed a group-based TMS (GTMS) to deal with compromised nodes effectively. Nevertheless, GTMS is not suitable for industrial applications since it imposes high communication overheads and memory overheads. Moreover, GTMS makes a decision by considering only communication trust that might be incorrect. GTMS uses static trust functions in which punishment and reward cannot be regulated according to application requirements. GTMS uses a week punishment coefficient and does not cover several security threats. In Jadidoleslamy et al.^19^, projected a fuzzy-based TMS (DTMS) based on SNs interactions to improve the decision-making capability of the trust evaluation process. The authors state that DTMS is scalable, precise, accurate, and demonstrates rapid convergent. Moreover, it can forecast trustworthiness. However, no mathematical validations are provided in support of its effectiveness for the contribution as mentioned above. In Singh et al.^20^, investigated a cluster-based trust assessment framework (LWTM) to diminish several internal attacks such as a whitewashing attack, node-capturing attack, and bad-mouthing attack. LWTM employs only communication trust to assess the reliability of SNs. Moreover, a priority concept along with a dynamic trust updating mechanism is used with static reward and punishment parameters. Considering only a single trust metric leads to incorrect trust decisions and makes it impractical for industrial applications. In Talbi et al.^21^, investigated a cluster-based adaptive trust model for WSN known as ADCT, which employs data trust and communication trust to deal with malicious attacks. The exponential and adaptive communication trust evaluation function of ADCT evaluates the activities of neighboring SNs. The data trust function discards fake recommendations of faulty SNs using statistical dispersion before indirect trust calculation. Experimental results illustrate its efficiency in terms of enhancement in cooperation among SNs with reduced overheads. However, while ADCT achieves good collaboration among SNs with minimal overhead, it ignores various important parameters such as energy trust, weight, and frequency of misbehavior, and hence it is not appropriate for on–off attack and real-time applications. In Xiang et al.^22^, projected a self-recommendation-based trust model for WSNs to perk up resource efficiency, load balancing, and malicious nodes detection capability. However, adequate evidence is not provided to demonstrate its effectiveness in terms of malevolent attack detection. It is complex, not viable, and undependable since it does not fulfill the fundamental requirements of WSNs. Saidi et al.^23^ investigate a secure and trustworthy CH election mechanism based on multiple metrics (trust, distance, and energy) and a misbehavior detection approach. If the CH is compromised, then a local clustering algorithm and cluster member level trust calculation scheme are adopted. Moreover, the behavior of SNs is monitored to remove the malicious SNs from the network since the removal of such nodes improves the throughput and lifespan of a sensor network. The performance is evaluated using MATLAB in the presence of malicious nodes. The proposed scheme can detect malicious nodes with few false alarms.

Kim et al.^24^ proposed a blockchain-based trust evaluation scheme to improve cooperation among nodes and eliminate spiteful nodes from the sensor network. The trust evaluation scheme considers the data trust and behavioral-based trust to obtain the trust score of nodes. Anwar et al.^25^ proposed a well-organized belief-based trust evaluation mechanism (BTEM) that identifies the malevolent node from reliable nodes and defends against diverse insider attacks such as on–off, DoS, and Bad-mouth attacks. BTEM utilize the concept of direct trust and indirect trust using the Bayesian estimation approach. The experimental results show better performance in the detection of spiteful nodes with minor delay and enhanced network throughput. In Guo et al.^26^, present a mutual evaluation-based lightweight clustered for WSNs. It is a linear time trust model employing multi-dimensional trust attribute to identify selfish entities, but it suffers from various limitations such as not being robust against collusion attack, on–off attack, and garnished attack. Moreover, it is not advisable for real-time applications since it does not employ misbehavior components, energy, and data trust. In Karthik et al.^27^, suggest a novel idea (HTMS) for accurate and reliable trust decisions in WSNs based on data quality as well as communication trust. HTMS employs provenance data, correlations metrics, flexible punishment, and reward to make it robust against selfish behaviors. Although HTMS incurs fewer overheads, it cannot alleviate several security threats such as collusion attack, Sybil attack, and on–off attacks since it does not consider the weight and frequency of unexpected behavior. In Firoozi et al.^28^, suggest a subjective-logic scheme to reduce redundant data as well as minimize resource consumption in static WSNs. The author employs the sliding window concept, correlation metrics, SNs locations, and their observed data to compute the trustworthiness of SNs. In Khan et al.^29^, proposed a realistic cluster-based trust model (LTS) to mitigate various internal attacks. LTS employs communication and data trust to make a correct trust decision. Moreover, it is adaptive and flexible since trust values can be tuned in accord with application requirements. However, LTS does not consider energy trust as well as the frequency of misbehavior and hence not effective in mitigating On–Off attack that makes it unrealistic for real-time critical industrial/business applications such as health care, safety, industrial machine, and defects monitoring, as well as controlling.

In Yang et al.^44^, proposed “An Intelligent Trust Cloud Management Method for Secure Clustering in 5G enabled Internet of Medical Things (IoMT)” to achieve reliable and secure communication. The proposed scheme constructs the standard trust clouds by employing an active training mechanism. Then fuzzy based trust computation of IoMT devices is initiated. After the trust assessment phase, an efficient trust classification scheme is employed to filter the malicious nodes. Finally, a trust cloud update mechanism is used to update the trust score of IoMT devices. The authors did not focus on the misbehavior component to improve reliability and cooperation.

In Su et al.^45^, investigated “A Redeemable Support Vector Machine-Dempster-Shafer (SVM-DS) Fusion-Based Trust Management Mechanism for Underwater Acoustic Sensor Networks” to achieve precise trust score of SNs and correct decision-making about any SNs. The proposed scheme (SVM-DS) considers three trust metrics: energy-based evidence, packet-based evidence, and data-based evidence. Then based on the above three pieces of evidence, SVM classify the trust score of SNs, and DS evidence theory is used to fuse the different trust classification results of SNs. Finally, trust redemption process and trust update mechanisms are employed to improve the accuracy of the trust computation process.

In Islambouli et al.^51^, investigated “Towards Trust-Aware IoT Hashing Offloading in Mobile Edge Computing” to provide competent as well as reliable distribution and offloading of hashing computation by formulating the distribution model as an integer linear programming problem. The investigated scheme considers the entropy-based direct opinion and indirect opinion trust computation as well as a time delay to solve the trust mechanism for IoT devices.

In Samir et al.^52^, investigated “Online altitude control and scheduling policy for minimizing Expected Weighted Sum AoI (EWSA) in UAV-assisted IoT wireless networks” by formulating a hard optimization problem. We formulate the IoT-UAV-BS status update problem as a Markov Decision Process (MDP) and develop deep reinforcement learning (DRL) to learn environment dynamics to handle the altitude and scheduling policy of Unmanned Aerial Vehicles. In particular, we leverage the Proximal Policy Optimization DRL stability algorithm to minimize EWSA.

The existing trust management schemes ^10–25,44,45,51,52^ failed to fulfill the most fundamental requirement for industrial WSN (ICN). Finally, after sincerely analyzing existing work, we can say that without considering indirect (feedback or reputation) trust, frequency of misbehavior, current, and past misbehavior, a malicious node might disguise the network to ruin its reputation^38^ and remain not detected as well as trustworthy^46–50^.

### Motivation

SNs-assisted ICNs have the ability to monitor (remotely) and control physical environments^2^. The survival of ICNs is highly dependent on the successful cooperation of tamper-resistant SNs^3^. Due to the aforementioned characteristics (e.g., wireless media, tamper-resistant SNs) ICNs are prone to various external and internal security threats^5^ that result in severe consequences for industry/business^8^. Unfortunately, most traditional cryptographic security mechanisms to achieve authentication, confidentiality, and integrity are not suitable to alleviate internal attacks^16^ in WSNs due to high implementation costs since they require high processing, high power consumption, and significant memory. TMSs provide a significant advantage over traditional cryptographic algorithms to improve security, resource efficiency as well as cooperation (dependability) among SNs against internal adversaries by estimating the trust level (score) of individual SNs^17^. A significant amount of research work has been carried out on trust modeling in the last decade but, to the best of our knowledge, existing work^17–25^, and^31–35^ did not fulfill the vital fundamental requirements (cooperation, resource efficiency, energy efficiency, algorithm complexity, coverage, connectivity, availability, high sensing fidelity, fault tolerance, data confidentiality, data integrity) for the survival of WSNs. Even most of the work^36–38^ has no adaptability, i.e., cannot be tuned according to application requirements and network capabilities. Furthermore, trust evaluation based on interactions^18^ monitored (detection of unexpected behavior) using a timing window^17^ is not reliable. There are other limitations in previous works such as the works in^40–42^ did not consider communication overhead and memory overhead while the ones in^42,43^ did not consider data trust. In^17^, the research did not consider energy trust as well as did not employ a flexible punishment coefficient. The works in^8–30^ did not employ weight and frequency of misbehavior. Moreover, they didn’t focus on an optimal number of clusters and suitable topology for WSN architecture.

### Applicability and implementation in real life scenarios

Computer systems are unable to observe various events (e.g. temperature, radiation) in the real world by themselves. Due to the advancement in wireless communications, WSN technology is an emerging concept used to sense the physical property of an event and convert it into a digital signal. With the worldwide emerging economies, various industries (oil, gas, automotive, utilities) are focusing on enhancing communication links between industrial sensor devices to ensure seamless communication using ICN. Since ICNs can operate in the harsh environment, many industries are adopting WSN infrastructure for industrial applications such as condition monitoring, environmental sensing, and process automation. The SNs monitor pressure, humidity, temperature, flow, level, viscosity, and density for processing, decision-making, and management. The processed data is transferred to a base station using intermediate SNs. In ICN, the SNs are linked wireless through different technologies such as ZigBee, Wi-Fi, Bluetooth, and WirelessHART. There are various challenges involved in ICNs such as security, reliability, and real-time response with lower latency. ICN must be able to examine the trust associated with routing messages between SNs, detect the presence of dangerous materials, and control the heating. Thus, ICNs require a functional and lightweight security system for better system performance that can detect faulty and malicious nodes through successful collaboration to achieve acceptable performance. The proposed secure, dependable trust assessment (SDTS) scheme for industrial WSNs shows their applicability since it provides robust security systems and secure routing by analyzing the data collected from the node´s behavior in real-time with lower latency. Furthermore, SDTS achieves high reliability with low complexity in an industrial environment by computing various trusts metric. However, the implementation of ICNs is application-specific. There are various concrete challenges during the implementation of ICNs such as real-time rescheduling, reliable and immediate message delivery, hardware constraints, latency communication resource constraint, integrated knowledge for IWSN applications, and centralized control architecture. However, the potential solution (SDTS) is used to improve security against internal attacks by cooperating with the SNs in all applications where security is a vital requirement. The real-time implementation using short-range transceivers and a low-cost 16-bit MSP430 processor is preferred since it minimizes energy consumption and implementation complexity. In real-life scenarios, ICNs can be used in hospital monitoring systems, process control, and automation of various industries and agriculture. There are various protocols (WLAN, WiFi, Bluetooth, Zigbee) but chosen of an appropriate protocol depends upon various parameters such as the number of nodes, Network range, and data size. Each SN can be implemented using MSP-EXP430G2 LaunchPad and the 868–870 MHz ISM band can be used to set up the ICN.

## Proposed work

In this research work, SNs are deployed with a hybrid (clustered) topology and a robust trust-based security protocol to enhance reliability, scalability, efficient resource allocation, efficient data routing, and system efficiency. SN trustworthiness is evaluated by monitoring its behavior using a watchdog mechanism^13^ to detect various kinds of attacks such as blackhole, selective forwarding, bad-mouthing, and DoS. Moreover, the concept of communication behaviors collection for trust estimation to the final trust determining for both the CMs and CHs is defined in^29^ in a detailed manner. Table 3 provides the list of abbreviations used in SDTS.

**Table 3:** List of abbreviations.

Abbreviation	Meaning
SNs	Sensor Node
WSN	Wireless Sensor Network
FPR	False-Positive Rate
*FNR*	False-Negative Rate
CPS	Cyber-Physical System
*BS*	Base Station
CH	Cluster Head
ICN	Industrial Communication Network
TAM	Trustworthy Assessment Models
TMS	Trust Management Scheme
MATLAB	Matrix Laboratory
MSP	Mixed Signal Processing
ISM	Industrial, Scientific, And Medical
CMs	Cluster Members
DoS	Denial-Of-Service
Opt	Optimal
fs	Free Space
mp	Multipath
T_max_	Maximum Trust Value
EWSA	Expected Weighted Sum Age-ofInformation
AoI	Age of Information

### Assumption

**Figure 1:**
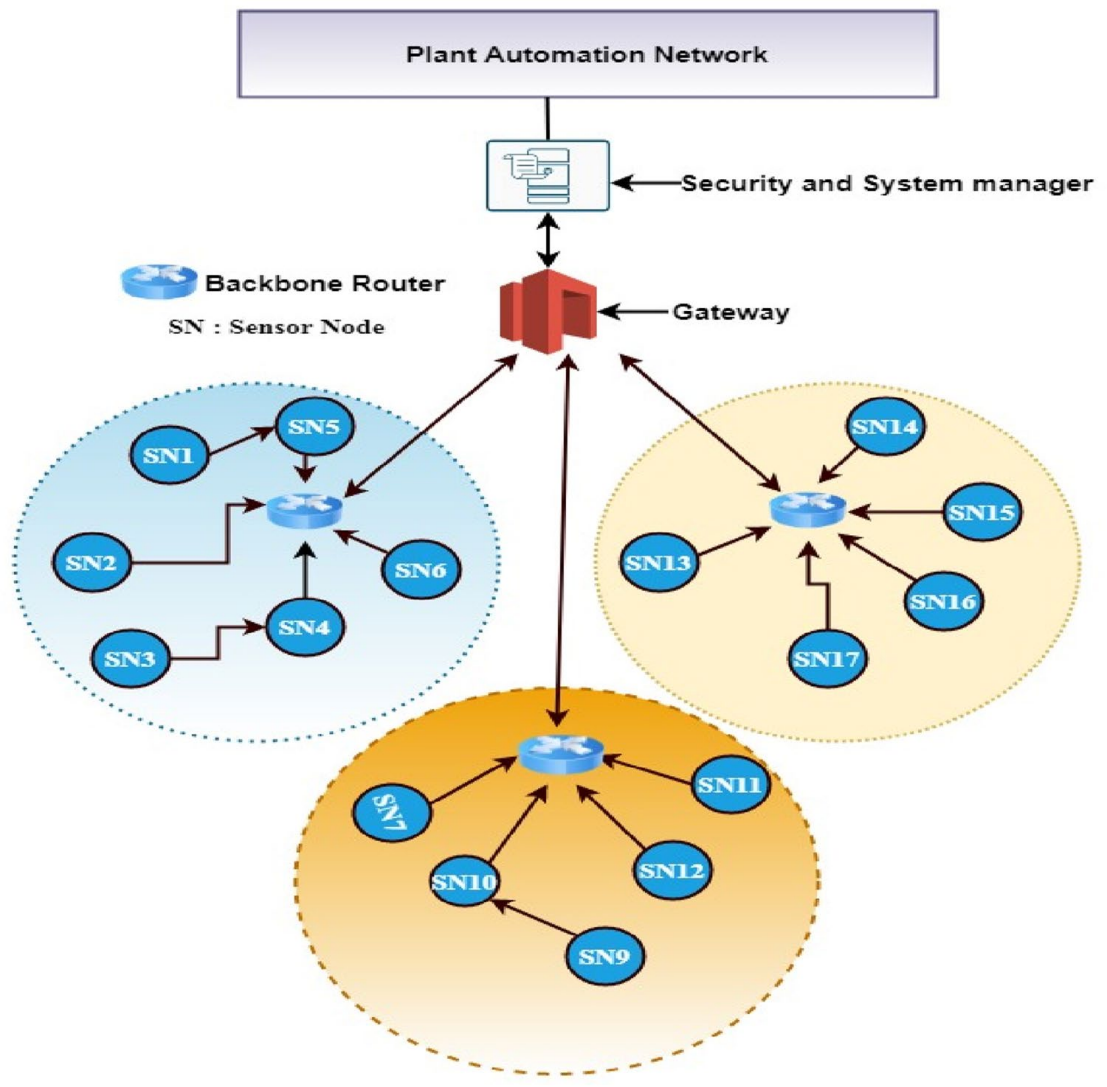
Clustered architecture of industrial sensor network.

For the following descriptions, please refer to Fig. 1.

**Sensor nodes**: After deployment, SNs are homogeneous and stationary. A unique ID is issued to each node. The stability, quick response, high sensitivity, and linear output of SNs make them suitable for detecting internal adversaries.

**Network initialization**: At the beginning of network initialization, the primary trust value of each node is assumed to be 10 (trustworthy). Moreover, we assume that all the SNs (field devices) have routing capability. The field devices monitor the industrial environment and forward/communicate the packets with other SNs, thus forming a multi-hop field network called ICN (IWSN). Nodes are considered to have data to communicate at all times, and data from neighboring nodes are correlated. Nodes are synchronized in real-time and have the ability to control power. The propagation channels are symmetric. The logical time window is defined in our previous work^29^ to record the good and bad interactions among SNs.

**Backbone Router**: The backbone router connects numerous field networks and acts as a link between them and the plant network. It works as a cluster head (CH). The CH dissipates the energy in three tasks, collection, aggregation, and transmitting the data. We presume that the CH is always a trusted SN.

**Gateway**: The gateway’s primary function is to translate network protocols and link IWSNs to the plant automation network. It works as a base station (BS).

**Network Manager**: The centralized network manager manages, monitor, and control the behavior of SNs without human intervention to improve the efficiency of the network by minimizing energy consumption with the help of the system manager and the security manager. Furthermore, the network manager allows the WSN to self-organize, and self-configure in case of failures without prior knowledge of the network topology. The system manager device is responsible for managing the network, devices, faults, and communications, while the security management system deals with security operations. The system manager and the security manager are merged into a single physical entity as shown in Fig. 1. The centralized security manager maintains the trust history of SNs and provides recommendations to improve the precision of the trust assessment process.

#### Optimal number of clusters

Dividing the entire WSN into an optimal number of clusters minimizes the overall energy consumption and improves network lifespan since network lifespan is dependent on the number of clusters. If we increase the number of clusters then the path used to forward sensitive data will contain maximum cluster heads resulting in high energy consumption. Furthermore, if the numbers of clusters are less than the distance between the SNs, CHs, and BS will increase which results in packet loss, high overhead on CH, and load balancing issues. Hence determining the optimal number of clusters is a vital step for the survival of WSNs. We consider a WSN with several SNs is indiscriminately deployed in a field, and it is divided into Kopt (optimal value of clusters) clusters. In this WSN, each individual cluster carries $N/Kopt$ SN, out of which one SN act as a CH and the remaining $(N/Kopt - 1)$ SNs are cluster member. As the BS is at the boundary, therefore, free spaces (fs) as well as multipath(mp) losses are considered. The optimal numbers of clusters depend upon the spatial distribution of SNs in the targeted field, distance between transmitting SN and BS as well as remaining energy in each SN. The optimal number of Clusters is obtained using Eq. (1) as follows.

1 $${k}_{opt}=\sqrt{\frac{{N}_{s}}{2\uppi }}*\sqrt{{\epsilon }_{fs}}*\frac{M}{D*(\sqrt{{\epsilon }_{mp}*{D}^{2}+{\epsilon }_{fs})}}$$

where D is the length of BS from CH. ${N}_{s}$ is total number of SNs divided into the clusters.

#### CM to CM trust evaluation scheme

During cluster member (CM) to cluster member (CM) trust evaluation, SNs are communicating with each other. The successful and unsuccessful communications are recorded in a logical time window^22^. Based on the recorded information in the logical time window, Eqs. (2) and (3) compute the direct trust values. Equation (2) is used to compute the direct communication trust and Eq. (3) is used to compute the direct data trust respectively.

2 $${T}_{x,y }\left(\mathrm{\Delta t}\right)=\left[{\mathrm{T}}_{\mathrm{max}}\times \left(\frac{{S}_{x,y}(\mathrm{\Delta t})}{\left({S}_{x,y}(\mathrm{\Delta t})+{q*U}_{x,y}(\mathrm{\Delta t})\right)}\right)* \frac{1}{\sqrt{{U}_{x,y}\left(\mathrm{\Delta t}\right)+1}}* {\upphi }^{{S}_{x,y}(\mathrm{\Delta t})}\right]$$

3 $${DT }_{x,y}^{D}=\left[{\mathrm{T}}_{\mathrm{max}}\times {( \frac{\mathrm{ S}{}_{\mathrm{x},\mathrm{y}}{}^{\mathrm{D}}\left(\mathrm{\Delta t}\right)+1}{\left(\mathrm{S}{}_{\mathrm{x},\mathrm{y}}{}^{\mathrm{D}}\left(\mathrm{\Delta t}\right)+\mathrm{q}*\mathrm{U}{}_{\mathrm{x,y}}{}^{\mathrm{D}}\left(\mathrm{\Delta t}\right)+2\right)})}^{\left(\frac{\mathrm{U}{}_{\mathrm{x,y}}{}^{\mathrm{D}}\left(\mathrm{\Delta t}\right)+1}{\mathrm{S}{}_{\mathrm{x,y}}{}^{\mathrm{D}}\left(\mathrm{\Delta t}\right)+\mathrm{q}*\mathrm{U}{}_{\mathrm{x,y}}{}^{\mathrm{D}}\left(\mathrm{\Delta t}\right)+2}\right)}\right]$$

The following algorithm 1 computes and updates the trust values at the intra-cluster level. In the proposed work, the symbol $\upphi$ is the reward factor, and $\theta$ represents the trust score threshold value. ${\mathrm{T}}_{\mathrm{max}}$ is the maximum trust value used in the research work. During experimental analysis, we chose 10 as ${\mathrm{T}}_{\mathrm{max}}\mathrm{ value}.$ We have already defined communication trust, data trust, indirect trust, energy trust, throughput, misbehavior trust, misbehavior component, dynamic slide length, and abnormal attenuation factor (q) in our previous work^13,14^.
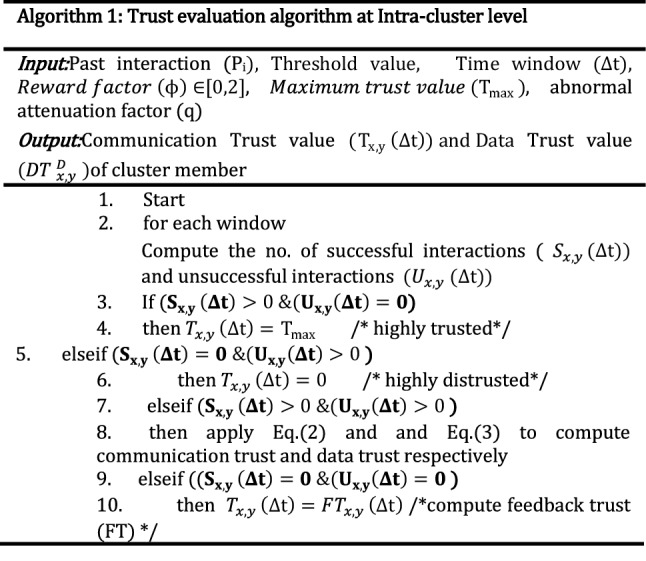


According to algorithm 1, when successful interactions between SN(x) and SN(y) are greater than zero and unsuccessful interactions are zero then the proposed system assigns maximum trust value (${\mathrm{T}}_{\mathrm{max}}$). On the other hand, when successful interactions between SN(x) and SN(y) are zero and unsuccessful interactions are greater than zero then the proposed system assigns zero trust value to SNs. Furthermore, when successful interactions between SN x and y are greater than zero and unsuccessful interactions are greater than zero then the proposed system assigns trust values according to the value computed by Eq. (2). When both successful (cooperative) communications and unsuccessful communications are zero, then the proposed system evaluates feedback (peer recommendation) trust to enhance the correctness of the proposed method in a hostile environment.

#### CM to CM peer recommendation Trust estimation ($\mathbf{FT}_{\mathbf{x,y} }\left({\varvec{\Delta}}\mathbf{t}\right))$

In peer recommendation trust evaluation, we only consider direct trusted neighbors to reduce communication overhead and improve the accuracy of TMS. The peer recommendation trust estimation improves the robustness of trust value since only direct trust value may be compromised by malicious SNs.The peer recommendation trust is calculated using Eq. (4).

4 $${PR}_{x,y }\left(\mathrm{\Delta t}\right) =\left[\left(\frac{{\sum }_{j=1}^{z}{T}_{x,j }\times {T}_{j,y}}{|z|}\right)\right]$$

where z is the set of directly trusted SNs. Here we don’t consider nodes having ${T}_{x,y }\left(\mathrm{\Delta t}\right)<\frac{{\mathrm{T}}_{\mathrm{max}}}{2}$ i.e., we eradicate malicious SNs to obtain robust trust values. Final trust value $({f}_{x,y}^{T}\left(\mathrm{\Delta t}\right))$ is computed by simply aggregating Eqs. (2)–(4)(simple averaging performs better than complex averaging) as shown by Eq. (5).

5 $$ f_{x,y}^{T} \left( {{\Delta t}} \right)) = \frac{{T_{x,y } \left( {{\Delta t}} \right) + { }DT _{x,y}^{D} { } + { }PR_{x,y } \left( {{\Delta t}} \right)}}{3} $$

In order to find the SNs status, ${FT}_{x,y }\left(\mathrm{\Delta t}\right) the$ component is used using Eq. (6) as follows.

6 $$ S\left( {FT_{{x,y~}} \left( {\Delta {\text{t}}} \right)} \right) = \left\{ {\left. {\begin{array}{*{20}l}    {\left[ {\left\lceil {\frac{{{\text{T}}_{{{\text{max}}}}  + 1}}{2}} \right\rceil ;\frac{{{\text{T}}_{{{\text{max}}}} }}{2}} \right]}  \\    {~~~\left( {0;\theta } \right)}  \\    {\left[ {\theta ;\left\lceil {\frac{{{\text{T}}_{{{\text{max}}}}  + 1}}{2}} \right\rceil } \right)}  \\   \end{array} } \right|\begin{array}{*{20}l}    {highly~\;trusted~\;node}  \\    {malicious\;~node~}  \\    {legitimate~\;node}  \\   \end{array} } \right\} $$

The value of $\theta$ is an application-dependent trust threshold whose value depends on application requirements.

#### CH to CH Direct trust estimation

The direct trust between cluster head i (CH_i_) and cluster head j (CH_j_) is computed in the same way as a cluster member level and defined by Eq. (7). The ${S}_{{\mathrm{CH}}_{\mathrm{i}}, {CH}_{j}}(\mathrm{\Delta t})$ represent the number of successful interactions between cluster head (i) and cluster head (j) at time $\mathrm{\Delta t}$. The ${U}_{{\mathrm{CH}}_{\mathrm{i}}, {CH}_{j}}(\mathrm{\Delta t})$ represent the number of unsuccessful interactions between cluster head (i) and cluster head (j) at time ${\Delta {\mathrm t}}$.

7 $$ T_{{{\text{CH}}_{{\text{i}}} , CH_{j} }} \left( {\Delta {\text{t}}} \right) = \left[ {{\text{T}}_{{\max }}  \times \left( {\frac{{S_{{{\text{CH}}_{{\text{i}}} , CH_{j} }} \left( {\Delta {\text{t}}} \right)}}{{\left( {S_{{{\text{CH}}_{{\text{i}}} , CH_{j} }} ^{{\left( {\Delta {\text{t}}} \right)}}  + U_{{{\text{CH}}_{{\text{i}}} ,~CH_{j} }} \left( {\Delta {\text{t}}} \right)} \right)}}} \right)*~\frac{1}{{\sqrt {U_{{{\text{CH}}_{{\text{i}}} , CH_{j} }} \left( {\Delta {\text{t}}} \right) + 1} }}*\phi ^{{S_{{{\text{CH}}_{{\text{i}}} , CH_{j} }} \left( {\Delta {\text{t}}} \right)}} } \right] $$

After computing the direct trust value at the inter-cluster level, the proposed system evaluates the indirect (feedback) trust through BS as follows.

#### BS to CH feedback Trust calculation

In order to obtain the CHs trust values, the BS periodically sends a request packet to cluster heads (suppose m) in the same fashion as the cluster head sends to cluster members. In response to the request packet, cluster heads send their direct trust values forwards to the base station. In order to compute feedback trust value, the BS maintains these values into a matrix using Eq. (8) as follows.

8 $$ B = \left[ {\begin{array}{*{20}c} {CH_{1,1} } & {CH_{1,2} } & {...} & {CH_{1,m} } \\ {CH_{2,1} } & {CH_{2,2} } & {...} & {CH_{2,m} } \\ {...} & {...} & {...} & {...} \\ {CH_{m,1} } & {CH_{n - 1,2} } & {...} & {CH_{m,m} } \\ \end{array} } \right] $$

The feedback trust value can be calculated by employing an extended (enhanced) beta distribution function using Eq. (9) as follows

9 $$ FT_{{BS,CH_{s} }} \left( {{\Delta t}} \right) = {\text{T}}_{{{\text{max}}}} \times \frac{{{\text{p}} + 1}}{{{\text{p}} + b + 2}} + \left[ {\left( {\frac{{\mathop \sum \nolimits_{r = 1}^{Q} T_{{{\text{CH}}_{{\text{r}}} ,{ }CH_{s} }} }}{\left| Q \right|}} \right)} \right] $$

where ${\mathrm{T}}_{\mathrm{max}}\mathrm{ is the maximum trust value}.\mathrm{ The symbol}$ p is positive feedbacks, b is negative feedbacks; Q is a total number of feedbacks of CHs towards CH j. A global trust value $({G}_{{CH}_{i},{CH}_{j}}^{T}\left(\mathrm{\Delta t}\right))$ can be obtained at CHs using Eq. (10) as follows

10 $$ \left( {G_{{CH_{i} ,CH_{j} }}^{T} \left( {\Delta {\text{t}}} \right)} \right) = \frac{{\alpha *~T_{{CH_{i} ,~CH_{j} }} \left( {\Delta {\text{t}}} \right) + \beta *~FT_{{BS,CH_{j} }} \left( {\Delta {\text{t}}} \right)}}{{\alpha  + ~\beta }} $$

where $\mathrm{\alpha and \beta }$ are respective weights $\mathrm{\alpha }+\upbeta$=1 and depending upon the application requirement, $\mathrm{\alpha and \beta }$ will give more flexibility to select appropriate weightage for the robust TMS. Where c is a positive constant that can be tuned according to TMS.

$$ {\upalpha } = {\text{s}}^{{\text{c*U}}} \;{\text{and}}\;{\upbeta } = 1 - {\text{ s}}^{{\text{c*U}}} . $$

## Simulation and result analysis

**Figure 2:**
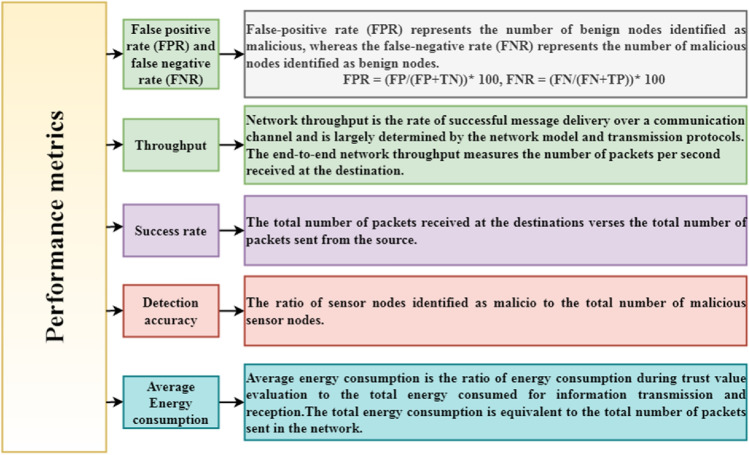
Performance metrics.

This section discusses the performance analysis of the proposed scheme (SDTS) using the MATLAB simulator. We consider two varieties of SN: good and malicious SNs. Good SNs cooperate with other nodes and behave well, but malicious SNs launch several internal attacks such as grayhole attacks, blackhole attacks, flooding, etc. Table 4 provides the list of parameters used in the simulation to obtain the experimental results. Performance metrics and evaluation is defined using Fig. 2. In this paper, we compare our SDTS with the latest schemes in^23,24^, and BTEM^25^. The main reason behind the selection of the comparison algorithm is the multiple interesting trust metrics used in these papers. Moreover, comparison algorithms are based on different techniques such as^24^ is blockchain-based and^25^ is a belief-based trust evaluation scheme.^23^ is based on trust, distance, and energy while^24^ is based on data trust and behavioral-based trust.^25^ BTEM utilizes the concept of direct trust and indirect trust using the Bayesian estimation approach. Simulation results exhibit that all three comparison algorithms provide acceptable results in harsh environments. For each scenario discussed in the results, we take some SNs as attacker nodes that perform various misbehavior. We steadily raise the misbehavior rate from 5% up to 50% and add various types of misbehavior nodes^13,14^ into the network. The aim is to measure various performance metrics.

**Table 4:** Simulation parameters.

Simulation parameters	
WSN size (Sensing area)	100∗100 m^2^
Network topology	Random
Network connectivity	25 m
Total SNs (n)	100–500
Sybil ID’s (SaNs)	2–7
No of rounds	100
Malicious nodes (M)	5–50%
Number of CHs	2–7
Transmission range	25 m
Optimal number of clusters	10–50
Range of cluster head	25 m
Distance among CHs	40 m
Each simulation iteration	100
Range of trust values	[0 10]
Initial trust value	10
Initial energy of SNs	0.5 Joule
Value of abnormal attenuation factor ($\mathrm{q}$) and forgetting factor (f)	$(0.5, 1]$
Trust threshold $(\theta )$	5
Packet size	50 bytes

**Figure 3:**
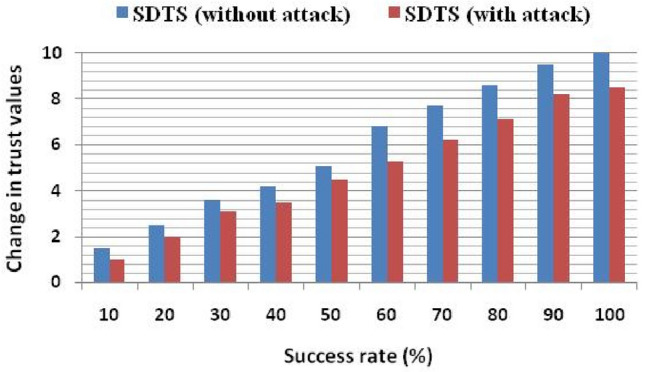
Change in trust values wrt. Success rate.

Figure 3 shows the effect of the success rate on the trust values. It shows that the trust value is 1.5 and 1 in normal (without attack) and malicious environments, respectively, with a 10% success rate. To analyze the performance of SDTS in a malicious environment, we have injected some malicious SNs at the 10th round in the network. These malicious nodes perform various misbehaviors such as packet dropping, abnormal energy consumption, and transmitting faulty data to other SNs, performing multiple attacks, and selfishness and behavior changing. Trust functions reduce the trust value of such malicious nodes. If SNs perform better and cooperate with each other, then trust value increases. With a 100% success rate, the trust value in a normal scenario is 10, while in the malicious environment, it is 8.5 since malicious SNs perform misbehavior with data as shown in Fig. 3. The trust level of the proposed SDTS system is increasing with the rise in success rate since the SDTS correctly analyze false report and accurately identify the malicious SNs.

**Figure 4:**
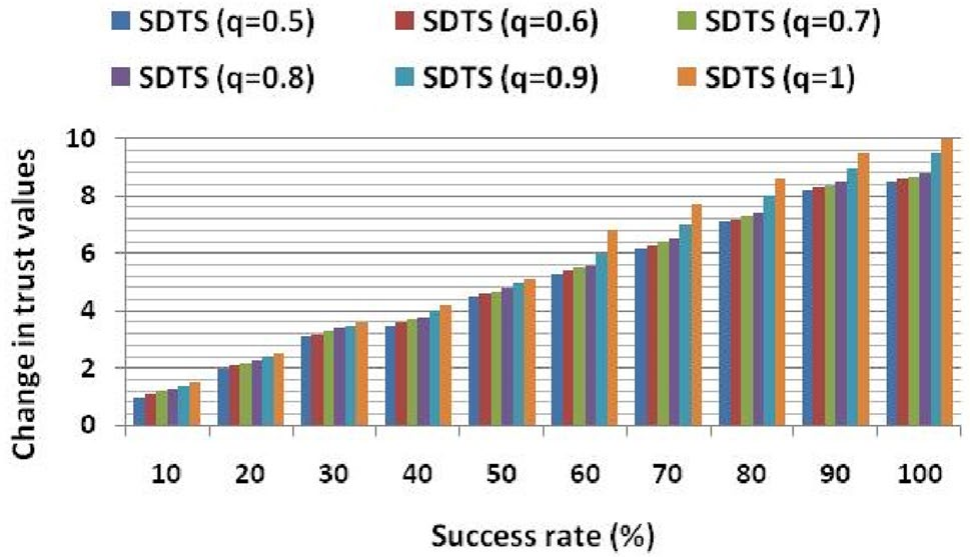
Change in trust values at different values of q.

Data trust (using Eq. (3)) checks the data consistency and detects misbehavior. Our scheme quickly reacts to misbehavior and decreases the trust value of malicious SNs. We also evaluate the trust value at different values of q in Fig. 4. It shows that as the success rate increases and the value of q increases, the trust value is also growing. Using the parameter q, we can reflect the effect of natural calamities on trust values. The existing schemes^23–25^ ignore such parameter and employ weak trust evaluation functions. Moreover, the methods^23–25^ do not show the success rate's effect on trust values in normal and malicious environments.

**Figure 5:**
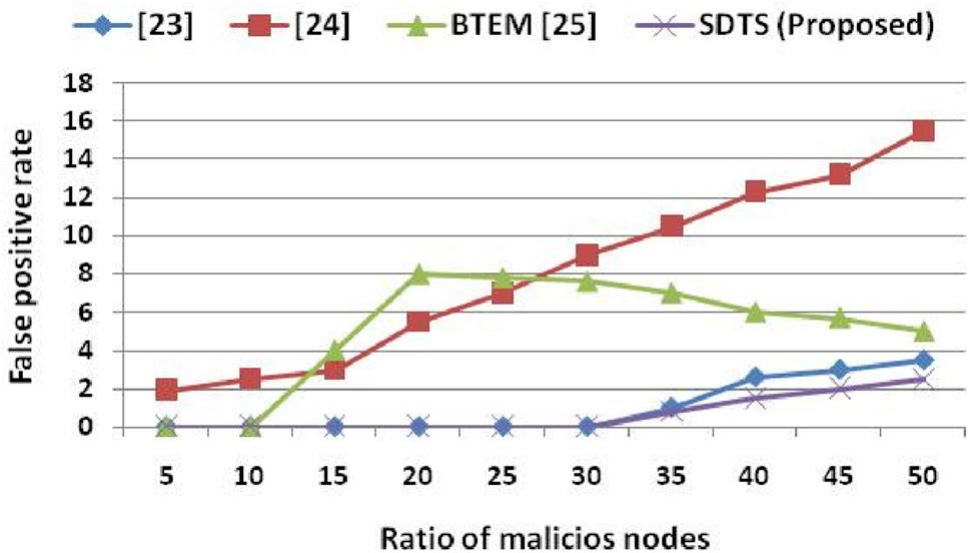
Ratio of malicious nodes vs. false-positive rate.

Figure 5 shows that the false positive rate (FPR) in SDTS remains comparatively low against the other schemes^23–25^as the ratio of malicious SNs (attacks) increases. The method to compute the performance metrics is shown in Fig. 2. Figure 5 shows that the false positive rate in^23^ is 0% up to 30% of malevolent SNs in a WSN consisting of 500 nodes. When we increase malicious nodes up to 35%, the FPR is 1%. When 50% of malicious nodes exist in the network, the FPR in^23^ rose to 3.5%. In^24^, the FPR is the highest than the other approaches due to non-adaptive trust functions being used to evaluate the reliability of SNs. In our proposed scheme (SDTS), the FPR is the lowest compared to^23–25^ since it incorporates cooperative interaction-based multi-trust and non-cooperative interaction-based trust. With 35% malicious SNs, the FPR of SDTS are 20%, 92.38%, and 88.57% lesser than^23–25^ respectively. Subsequently, with 50% malicious SNs, the FPR of SDTS are 28.57%, 83.87%, and 50% lesser than^23–25^, respectively. Since the malicious nodes are caught and removed by the proposed strategy, the FPR is reduced.

**Figure 6:**
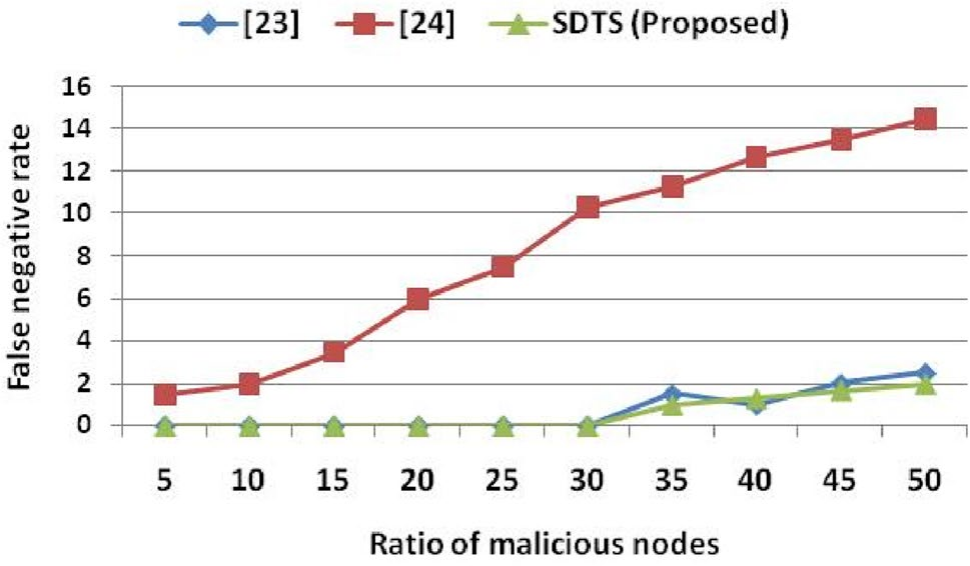
Ratio of malicious nodes vs. false negative rate.

The misbehavior component in the algorithm (weight of misbehavior and frequency of misbehavior) eliminates those malicious SNs that change their behavior as sometimes good and sometimes bad. If nodes are frequently interacting, then cooperative interaction-based trust evaluation detects the malicious behavior of SNs and eliminates these SNs to reduce the FPR. Whenever the success rate is low, i.e., nodes are rarely interacting, then the non-cooperative interaction-based trust evaluation computes the rate of misbehavior, the weight of misbehavior, aggregate misbehavior, and frequency of misbehavior using our previous work^14^ to eliminate such malicious nodes to reduce the FPR. The non-cooperative interaction-based trust evaluation mainly detects on–off nodes that change their behavior frequently. Whenever SNs change their behavior, the misbehavior component helps to detect such SNs. Other schemes^23–25^ do not consider the misbehavior component hence fail to detect the on–off attack. The behavior of SNs is recorded in the logical time window used in our previous work^22^. Similar to the FPR, the false-negative rate (FNR) of SDTS is lesser than the results in^23^ and^24^. Since the existing work^25^ does not provide any result in terms of FNR, we only compare SDTS with^23^ and^24^ in Fig. 6. Figure 6 shows that SDTS achieved reduced FNR in a network consisting of up to 50% of malicious SNs. In SDTS, the FNR is 0% up to 30% of malicious SNs. Moreover, the FNR is 1%, 1.3%, 1.7%, and 2% in a network consisting of 35% malicious SNs, 40% malicious SNs, 45% malicious SNs, and 50% malicious SNs, respectively. The FNR in SDTS is 33.33% and 91.15%, lesser than^23^ and^24^, respectively. Moreover, in 50% malicious SNs condition, the FNR of SDTS is 20% and 86.20% lesser than^23^ and^24^, respectively. The misbehavior component in non-cooperative interaction-based trust evaluation plays a vital role in reducing the FPR and FNR in the network of malicious SNs.

**Figure 7:**
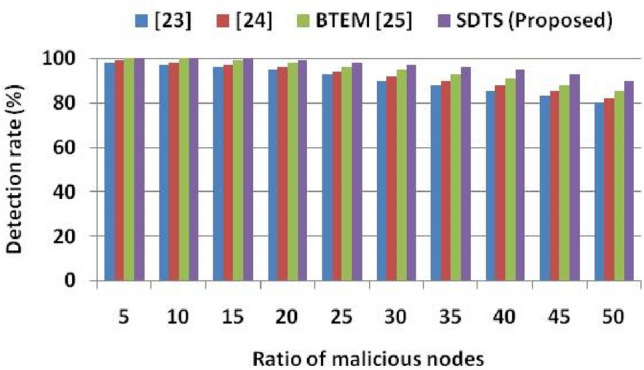
Ratio of malicious nodes vs. detection rate.

Figure 7 shows the malicious SNs detection rate in a network of 500 SNs. We inject up to 50% malicious behavior to determine the performance of SDTS in harsh environments. Malicious nodes transmit faulty data to other nodes, including CH and BS. After simulation, we found that when 5% malicious SNs are in the network,^23^ can detect 98% of malicious SNs,^24^ can detect 99% of malicious SNs,^25^ and SDTS can detect 100% of malicious SNs. When 15% malicious SNs are in the network,^23^ can detect 96% of malicious SNs,^24^ can detect 97% of malicious SNs,^25^ can detect 99% of malicious SNs, and SDTS can detect 100% of malicious SNs. When 30% malicious SNs are in the network,^23^ can detect 90% of malicious SNs,^24^ can detect 92% of malicious SNs,^25^ can detect 95% of malicious SNs, and SDTS can detect 97% of malicious SNs. When 50% malicious SNs are in the network,^23^ can detect 80% of malicious SNs,^24^ can detect 82% of malicious SNs,^25^ can detect 85% of malicious SNs, and SDTS can detect 90% of malicious SNs. SDTS can detect 12.5% more malicious SNs than^23^, 11.11% more malicious SNs than^24^, and 5.88% more malicious SNs than^25^ in a WSN consisting of 50% malicious SNs since SDTS detect misbehavior at an earlier stage during communication trust and data trust using efficient trust functions. The early detection and separation of malicious SNs saved the energy, bandwidth, and transmission power required for the re-transmission of data packets.

**Figure 8:**
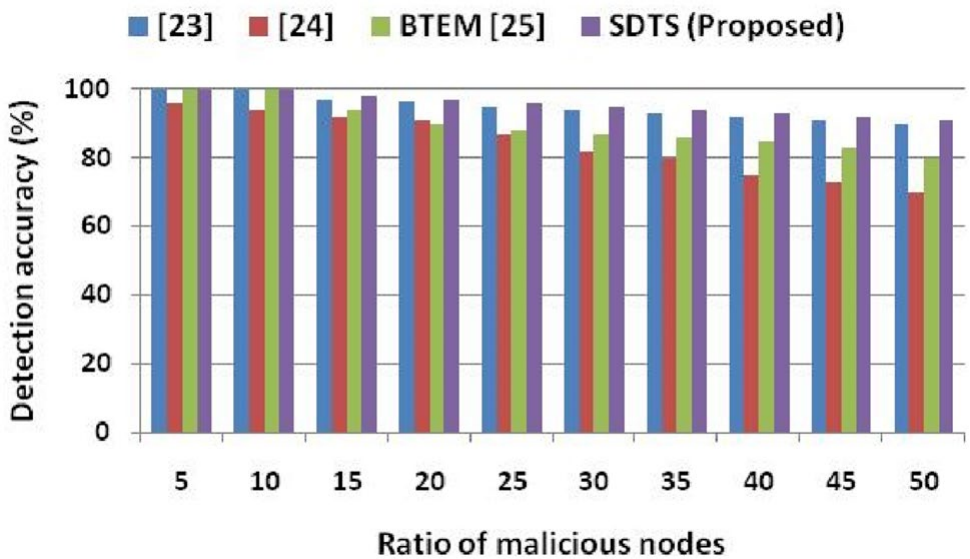
Ratio of malicious nodes vs. detection accuracy.

Once the misbehavior is detected, SDTS decreases the trust value of that particular SN. SDTS improves the trust score of SNs only if SNs qualify the communication trust, data trust, and indirect trust. SDTS considers the misbehaviors of SN at any round and decreases its trust value. On the other hand, the detection accuracy of SDTS is higher than^23–25^, as shown in Fig. 8. However, detection accuracy decreases as the ratio of malicious SNs increases since much false information is available in the network. We examine the detection accuracy by performing several rounds of stimulation consisting of 500% nodes and up to 50% malicious SNs.

**Figure 9:**
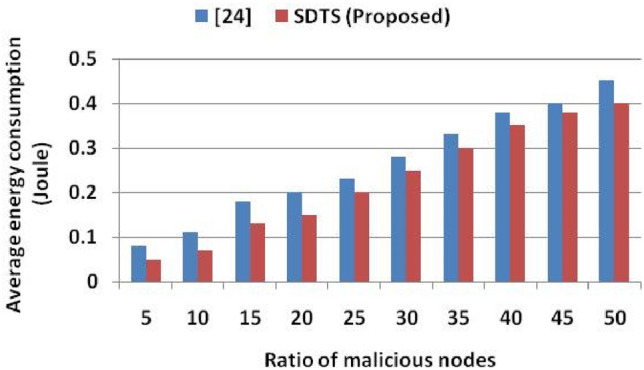
Ratio of malicious nodes vs. average energy consumption.

We observe that the detection accuracies of^23–25^ and SDTS are 100%, 96%,100%, and 100% when 5% SNs are malicious in the WSN. With 10% malicious SNs in the WSN, the detection accuracies of^23–25^ and SDTS are 99.9%, 94%, 99.9%, and 99.9% respectively. With 25% malicious SNs in the WSN, the detection accuracies of^23–25^ and SDTS are 95%, 87%, 88%, and 96% respectively. Moreover, with 50% malicious SNs in the WSN, the detection accuracies of^23–25^ and SDTS are 90%, 70%, 80%, and 91% respectively. SDTS improves 1.1%, 23.07%, and 12.08% than^23,24^, and^25^ in terms of detection accuracy when WSN consists of 50% malicious SNs. The reason behind this remarkable performance of SDTS is the efficient and adaptive trust function that improves dependability and defeats malicious SNs. SDST employs an efficient strategy to identify the status of SNs depending on the success ratio and interaction frequency. If SNs interact a sufficient number of times within the time window period, we compute communication (direct, indirect) trust and data trust. Furthermore, we check the deviation degree, data rate delivered, and data consistency using^13,14^ to identify the malicious SNs. Figure 9 shows the average energy consumption in the presence of malicious nodes. We assume that each packet forwarding requires 0.001-J energy and increases the temperature of SNs by 0.1 units. We compare average energy consumption with^24^ since the approaches in^23^ and^25^ do not provide any result in terms of average energy consumption. Figure 9 shows that the average energy consumptions in^24^ and SDTS are 0.08 J and 0.05 J, respectively, with 5% malicious nodes. Then, the average energy consumptions in^24^ and SDTS are 0.18 J and 0.13 J, respectively, with 15% malicious nodes. After that, the average energy consumptions in^24^ and SDTS are 0.28 J and 0.25 J, respectively, with 30% malicious nodes. Furthermore, the average energy consumptions in^24^ and SDTS are 0.45 J and 0.40 J, respectively, with 50% malicious nodes.

**Figure 10:**
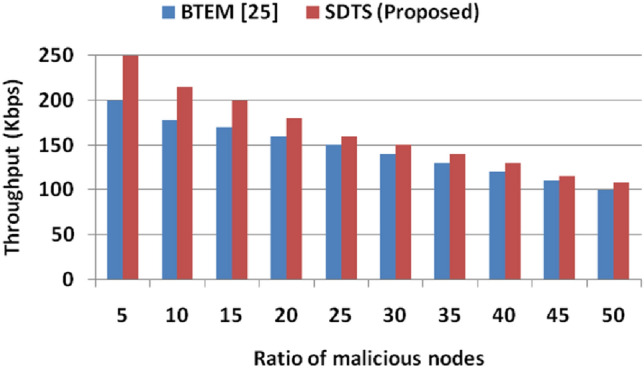
Ratio of malicious nodes vs. throughput.

SDST consumes 11.11% less energy than^24^ in a malicious environment consisting of 50% selfish SNs due to improved dependability level and smaller energy consumption among SNs, while the other methods demonstrate elevated energy consumption due to increased overhead and communication cost. Figure 10 shows the comparative throughput (kbps) in the presence of malicious SNs. We compare the throughput against that of^25^ since^23^ and^24^ do not provide throughput analysis in their results and discussion section. SDTS achieves better throughput than^25^ since SDTS discourages malicious SNs participation in the WSN. Since the malicious nodes are detected quickly and timely in our proposed approach, the throughput is higher than that of^25^. SDTS throughput is also higher than^25^ because the SDTS considers both the dependability and energy level of the SNs. With 5% malicious nodes in WSN, the throughputs of^25^ and SDTS are 200 kbps and 250 kbps, respectively. With 10% malicious SNs, the throughputs of^25^ and SDTS are 178 kbps and 215 kbps, respectively. Moreover, with 20% malicious SNs, the throughputs of^25^ and SDTS are 160 kbps and 180 kbps, respectively. Furthermore, with 50% malicious SNs, the throughputs of^25^ and SDTS are 100 kbps and 108 kbps, respectively, which is 8% superior to^25^.

## Conclusion and future direction

This paper proposes a secure and dependable trust assessment scheme (SDTS) for industrial communication networks to improve the dependability and cooperation among SNs. SDTS improves security in IWSNs by introducing an efficient trust model that detects malicious nodes and improves the trust system's accuracy. In SDTS, first, we divide the networks into the optimal number of clusters to improve the survival of IWSNs. After dividing it into the optimal number of clusters, SDTS computes direct communication trust, data trust, and indirect trust with the help of the records maintained in a logical time window. Moreover, SDTS incorporates abnormal attenuation factors and dynamic slide lengths to deal with various natural calamities and internal attacks. During the non-cooperative interaction interaction-based trust evaluation process, we compute the aggregate misbehavior, rate, and weight of misbehavior, frequency of misbehavior, and final misbehavior-based trust function. The simulation of SDTS is performed using MATLAB 2019(a), and the results are obtained in terms of change in trust value, false-positive rate (FPR), false-negative rate (FNR), attack detection rate (%), detection accuracy (%), average energy consumption (joule) and throughput (kbps). In the network consisting of 50% malicious SNs, the FPR of SDTS are 28.57%, 83.87%, and 50% lesser than^23,24^, and BTEM^25^, respectively. Moreover, in 50% malicious SNs, the FNR of SDTS are 20% and 86.20% lesser than^23^ and^24^, respectively. Furthermore, SDTS can detect 12.5% more malicious SNs than^23^, 11.11% more malicious SNs than^24^, and 5.88% more malicious SNs than^25^ in a WSN consisting of 50% malicious SNs. Moreover, with 50% malicious SNs in the WSN, the detection accuracies of^23–25^ and SDTS are 90%, 70%, 80%, and 91% respectively. SDTS improves 1.1%, 23.07%, and 12.08% than^23–25^ in terms of detection accuracy when WSN consisting of 50% malicious SNs. SDST consumes 11.11% less energy than^24^ in a malicious environment consisting of 50% selfish SNs. Furthermore, with 50% malicious SNs, the throughputs of^25^ and SDTS are 100 kbps and 108 kbps, respectively, which is 8% superior to^25^.

In the future, we are planning to examine the communication overhead, scalability, and convergence time of the proposed SDTS. Moreover, we are planning to design a machine learning-based trust model for early forest fire detection using intelligent WSNs.

## Data Availability

The datasets generated and/or analyzed during the current study are available from the corresponding author on reasonable request.
